# STARNET 2: a web-based tool for accelerating discovery of gene regulatory networks using microarray co-expression data

**DOI:** 10.1186/1471-2105-10-332

**Published:** 2009-10-14

**Authors:** Daniel Jupiter, Hailin Chen, Vincent VanBuren

**Affiliations:** 1 Department of Systems Biology and Translational Medicine, College of Medicine, Texas A & M Health Science Center, Temple, TX 76504, USA

## Abstract

**Background:**

Although expression microarrays have become a standard tool used by biologists, analysis of data produced by microarray experiments may still present challenges. Comparison of data from different platforms, organisms, and labs may involve complicated data processing, and inferring relationships between genes remains difficult.

**Results:**

**STARNET 2 **is a new web-based tool that allows post hoc visual analysis of correlations that are derived from expression microarray data. **STARNET 2 **facilitates user discovery of putative gene regulatory networks in a variety of species (human, rat, mouse, chicken, zebrafish, *Drosophila*, *C. elegans*, *S. cerevisiae*, *Arabidopsis *and rice) by graphing networks of genes that are closely co-expressed across a large heterogeneous set of preselected microarray experiments. For each of the represented organisms, raw microarray data were retrieved from NCBI's Gene Expression Omnibus for a selected Affymetrix platform. All pairwise Pearson correlation coefficients were computed for expression profiles measured on each platform, respectively. These precompiled results were stored in a MySQL database, and supplemented by additional data retrieved from NCBI. A web-based tool allows user-specified queries of the database, centered at a gene of interest. The result of a query includes graphs of correlation networks, graphs of known interactions involving genes and gene products that are present in the correlation networks, and initial statistical analyses. Two analyses may be performed in parallel to compare networks, which is facilitated by the new **HEATSEEKER **module.

**Conclusion:**

**STARNET 2 **is a useful tool for developing new hypotheses about regulatory relationships between genes and gene products, and has coverage for 10 species. Interpretation of the correlation networks is supported with a database of previously documented interactions, a test for enrichment of Gene Ontology terms, and heat maps of correlation distances that may be used to compare two networks. The list of genes in a **STARNET **network may be useful in developing a list of candidate genes to use for the inference of causal networks. The tool is freely available at , and does not require user registration.

## Background

Expression microarrays have become a widely used platform for assaying the differences in the transcriptomes of two experimental settings. While the technology has gained wide acceptance, the analysis of the data produced from a microarray experiment may yet present challenges to experimentalists. This is the case both for array experiments performed in-house by individual labs, and for retrospective analysis of array experiments that have been conducted elsewhere. The problem is exacerbated when considering comparisons between different experiments, platforms, and model organisms. Basic analysis of microarray experiments typically produces lists of differentially expressed genes. The central challenge of basic microarray analysis is thus to ascribe biological meaning to the members of the list of differentially expressed genes by inferring the relationships between these genes and the relationships between the genes and the experimental milieu. These problems are of crucial importance given that experiments are costly and time consuming, and given that public-domain databases such as the Gene Expression Omnibus (GEO) [1,2] contain thousands of array experiments with potential for exploration by post hoc analysis.

A central motivation for creating the **STARNET **application was to leverage this tremendous resource of microarray data for the discovery of putative gene regulatory relationships and other biological interactions, prior to conducting additional costly wet lab experiments. This tool provides insights that may guide experimentation by fostering new hypotheses, or may provide additional support for previously formed hypotheses. The results may also be used to develop a preliminary list of genes to use as input for other regulatory network discovery and validation tools, such as those involving Bayesian inference or probabilistic Boolean networks.

Given a gene of interest provided by the user, **STARNET **mines precomputed correlations from a collection of microarray expression data, which we refer to hereafter as a data *cohort*, and builds a correlation network centered at that gene. The visual data is also presented as text and is supplemented by annotations that were retrieved from NCBI database tables.

A previous murine-only version of **STARNET**, which included both a full and developmental cohort of arrays, has been online since July 2007 [3]. The current effort 1) expands the coverage to ten different species, 2) allows cross-species comparisons, and 3) introduces a new tool, **HEATSEEKER**, for drawing false color maps comparing two selected networks. Additionally, the user interfaces for both **STARNET 2 **and its predecessor have been improved for greater ease of use, and the responses to user queries have been improved for better visual organization and navigation of the displayed results.

In this report we describe the construction and use of **STARNET 2**, describe the new **HEATSEEKER **module, and discuss the output produced by user queries. **STARNET **uses an approach that is uncommon in several ways. First, while there are numerous tools for the analysis of microarray data [4-12], there are relatively few tools that facilitate retrospective analysis or data mining of microarrays, e.g. [13]. Second, rather than attempt to identify differential gene expression for a narrow range of experimental questions, **STARNET **identifies gene pairs with high magnitude correlation across a large number of experiments, thus providing strong statistics that include confidence intervals. Third, although we have pre-selected the data cohorts for retrospective analysis, **STARNET **allows user control over the general size and topology of the networks produced, and performs an on-the-fly test of GO term enrichment for those networks, along with a database search of known interactions involving genes and gene products from the prescribed networks. Thus, while tools such as **STRING **[14] and **YEASTNET **[15] provide a data integration approach to assessing likely functional protein interactions, **STARNET **better facilitates exploratory analysis of selected data cohorts with finer control over general network size and topology. Moreover, previous approaches that have performed large-scale retrospective analyses have not always supplied a database for searching and reviewing their results, apart from supplying large data files as supplementary materials [16]. Finally, **HEATSEEKER **enhances the analysis provided by **STARNET **by allowing users to directly compare the networks produced by two different data cohorts, which includes a provision for comparing data from two different species. **HEATSEEKER **makes an unbiased comparison by combining the lists from both networks and then comparing only those genes that share orthologues on both platforms. **HEATSEEKER **will thus provide insight into the differential wiring of gene regulatory networks among different species. This combination of uncommon attributes marks **STARNET 2 **as a unique and powerful tool for accelerating discovery of gene regulatory networks.

## Implementation

Data collection and preprocessing was performed using procedures from Jupiter and VanBuren [3] that were slightly modified as described below. Briefly, for each organism represented, data was collected from between 148 (rice) and 3,763 (human) Affymetrix microarray samples (Table 1). These data were downloaded from NCBI's GEO. A total of 12,762 arrays were used in this analysis, which is approximately 5% of the samples in GEO (as of August 2008). Complete lists of array platforms used, and the experiments selected for our analysis are available at . Array probes were mapped to NCBI Gene [17] IDs using version 11 of the alternate mapping of Affymetrix chips provided by Dai et al. [18]. After the data were normalized using the **JUSTRMALITE **[19] normalization method implemented in the BioConductor [20] suite of tools for R, Octave was used to compute pairwise Pearson correlation coefficients between the expression patterns of the genes within each array platform. For human, rat, mouse and *Drosophila *we also computed correlations for a subset of arrays corresponding to development. We refer to these two sets of correlations, respectively, as the 'full' and 'development' cohorts. Computed correlations and Entrez Gene tables were combined in a new MySQL database, for easy access and manipulation. Further information from NCBI databases, including interactions from the Gene Reference Into Function (Gene RIF) files at NCBI's FTP site  were also loaded into the relational database.

**Table 1:** Expression microarray data represented in STARNET 2

**Species**	**Full Cohort Arrays**	**Development Cohort Arrays**	**Genes on Array**
*Homo sapiens*	3,763	372	17,726
*Mus musculus*	2,145	239	16,631
*Rattus norvegicus*	1,982	247	11,427
*Gallus gallus*	164	-	12,491
*Danio rerio*	222	-	6,838
*Drosophila melanogaster*	454	195	13,060
*Caenorhabditis elegans*	381	-	15,015
*Saccharomyces cerevisiae*	254	-	5,566
*Arabidopsis thaliana*	3,249	-	21,281
*Oryza sativa*	148	-	23,419

The second column is the number of arrays used in the full cohort, the third is the number of arrays used in the development cohort, and the fourth column indicates the number of genes on the array platform, according to the re-annotation by Dai and colleagues.

The set of correlation coefficients thus derived has a large memory footprint and contains a large amount of data that is of little interest from our perspective (i.e., low magnitude correlations). Thus, this collection was trimmed in a variety of ways. First, the 100,000 highest magnitude positive and negative correlations for each cohort were extracted. As highly correlated groups of genes in a correlation network exhibit a high amount of interconnectedness, or *cliquishness*, this distribution does not necessarily include all genes on an array. To guarantee full coverage, we constructed another sub-distribution through gene-by-gene extraction of the ten highest magnitude positive and negative correlations for that gene. This guarantees that each gene on the array is available for user queries. As described previously, other specialty distributions were also created, for more focused study on genes related to transcription and signaling [3].

Network construction algorithms were implemented in Perl. The user interface was built using Perl-CGI, and graphs are created on demand using the **GRAPHVIZ **package available from AT&T . **HEATSEEKER **false-color maps are created on demand using R/BioConductor.

## Results

On the **STARNET 2 **webpage  the user enters a gene of interest as either an Entrez Gene ID or gene symbol, and selects either one or two data cohorts to examine. The user selects how many network levels to draw (*l*), and how many connections are to be made per level (*n*). Connections are then drawn between the gene of interest (Level 0) and the *n *genes with the highest magnitude correlations of co-expression with the gene of interest (Level 1 genes). Connections are then drawn from the Level 1 genes to Level 2 genes, and so on, until *l *levels have been built as the user specified. Further options for network topology specification and alternate sub-distributions of correlations are available, and are detailed in the documentation available on the webpage, .

**Figure 1:**
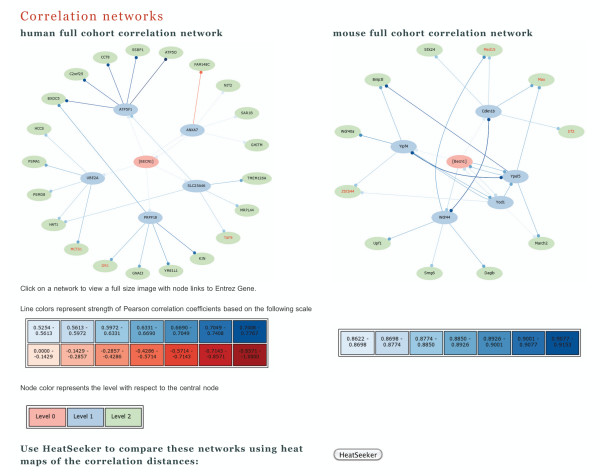
**STARNET 2 networks for *BECN1 *in the human and mouse full cohorts, using default settings**.

A graph of correlations is drawn for the specified gene for each data cohort that is selected. Lines connecting genes are color coded to indicate the magnitude of the correlations, with a scale provided below the graph. By default, genes annotated with Gene Ontology (GO) [21] terms containing the word "transcription" are highlighted in the network that **STARNET **draws. The user may elect to change or omit the search term. Genes common to both networks (or orthologous genes, in the case of cross-species comparisons) are highlighted. An example of the correlation networks generated by **STARNET 2 **is shown in Figure 1. These networks are constructed for the central gene *BECN1*, which was selected as a representative example, and are drawn using **STARNET 2**'s default settings from correlations computed in the human [Entrez Gene Symbol:*BECN1*, Entrez ID:8678] and mouse [Entrez Gene Symbol:*Becn1*, Entrez ID:56208] full data cohorts, respectively. Network images are linked to NCBI, so that a mouse-click on a gene node will redirect the user to the Entrez Gene entry for that gene.

To aid exploratory analysis of the networks, data is also presented in a tabular format. Lists of genes and correlations are provided, with links to the Entrez Gene entries for each gene. Genes common to both networks and those highlighted with the GO search term are also listed with appropriate hyperlinks to external sites.

Interpretation of the correlation networks is further facilitated by (a) drawing and listing networks of known interactions involving the genes in each correlation network, and by (b) performing a hypergeometric test of GO term enrichment for the genes within each network, relative to the entire complement of gene features on the array on which they were assayed. Enriched GO terms are provided together with lists of the genes annotated by the respective terms, and the terms are linked to AMIGO for detailed reference. As with the correlation networks described above, nodes in the documented interaction networks are linked to Entrez Gene.

Users may select any two of the available data cohorts for comparison, including comparisons between the 'full' cohort for an organism and that organism's 'development' cohort, as well as cross-species comparisons. This allows side-by-side comparison of the networks derived from orthologous genes in different species.

**Figure 2:**
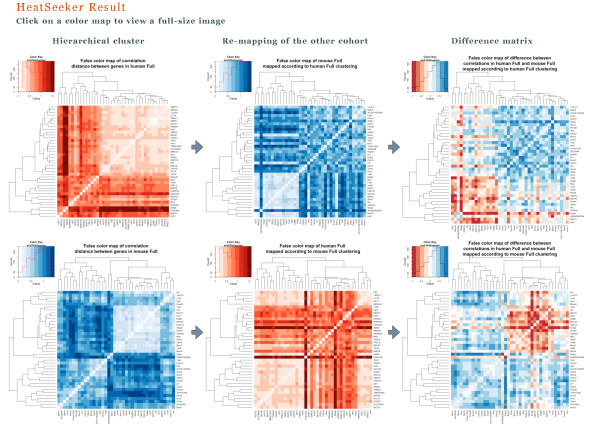
**HEATSEEKER results for the networks shown in Figure 1**.

**STARNET 2 **offers a newly developed module called **HEATSEEKER**, which draws false color maps that allow a direct visual comparison of the co-expression patterns from two networks. The union of the genes from both networks (or super-network), where orthologous genes that are on both array platforms are identified for cross-species analysis, is sent to the **HEATSEEKER **application when the user mouse-clicks the 'HeatSeeker' button on the **STARNET 2 **result page. **HEATSEEKER **draws a false color map of correlation distances between genes in the super-network for each cohort, where the color maps are arranged with complete-linkage hierarchical clustering. For each cohort's clustering, the other cohort is re-mapped using that clustering, and the resulting reordered color map is displayed. Finally, for each clustered color map and its re-mapped counterpart from the other cohort, **HEATSEEKER **draws a false color map of the difference between the correlations in the first and the second cohort. Figure 2 shows the **HEATSEEKER **result for the networks drawn in Figure 1. Individual heat maps may be mouse-clicked on the result page to reveal a full sized image. Tabular output of the data represented in the false color maps is also made available for download, where statistical significance of differences in the correlations at p ≤ 0.05 is indicated with '*', and statistical significance at p ≤ 0.01 is indicated with '**'.

Full documentation for **STARNET 2 **is available at .

## Conclusion

**STARNET **is a useful tool for discovery of putative gene regulatory networks. Such efforts are facilitated by the graphs of known interactions of genes and gene products that are supplied together with the correlation networks produced by **STARNET**. Known interactions are sometimes reflected within the correlation networks produced by **STARNET**, which supports the biological relevance of these networks. **STARNET **may thus be used to suggest new lines of research. Graphical depictions of data often supersede the utility of the same data presented in a table.

The notion of using correlations between the expression profiles to foster insight into gene function is neither contentious nor novel. However, in future studies it will be useful to assess **STARNET **from a quantitative perspective to evaluate its ability to recapitulate segments of known biological networks [22]. This is an important area of inquiry, as it will give some insight about the extent to which edges in **STARNET **correlation networks may be used to predict regulatory relationships.

Recent efforts have suggested the utility of measuring changes in correlation as an important complement to measuring differential expression in microarray experiments, as changes in correlation are indicative of differential wiring of regulatory networks [3,23,24]. In the first version of **STARNET**, differential wiring could be crudely assessed between a correlation network built from heterogeneous data sets, and a correlation network derived from a smaller subset of the data related to mouse heart development. With the cross-species capabilities introduced in **STARNET 2**, users may now consider using knowledge of one species to supplement knowledge of regulatory networks in other species, and may use **STARNET 2 **to develop new hypotheses regarding differential wiring between species, and for four of those species, between a large heterogeneous data set and a smaller data set related to development. Additionally, the **HEATSEEKER **module is a first step in towards a more careful and unbiased comparison of the networks derived from two different data cohorts.

**STARNET 2 **presents an intuitive, fast, and free way to produce preliminary impressions of gene regulatory relationships. Other methods for similar types of analysis are available. For example, clustering methods [4,25-27] offer a simple way to group genes into modules of (potentially) interacting and interrelated genes. These results are qualitative, and lack any indication of how interactions within a module occur. At the other extreme, methods involving ordinary differential equations offer a much higher resolution view of regulatory networks. However, these methods require some preliminary knowledge of the network being modeled. Lying between these extremes, Bayesian networks [28-33] provide both qualitative and quantitative data. This class of techniques is both theoretically and computationally expensive, and often employs heuristics to obtain the networks. These approaches also typically require time series data. **STARNET 2 **offers an attractive alternative: it produces both qualitative and quantitative data using a straightforward methodology that is highly accessible to experimental biologists. Furthermore, the default settings of **STARNET 2 **will generate a list of correlated genes that is ≤ 31 genes, and such lists may be a useful starting place for inferring causal networks using one the other methods mentioned above, such as Bayesian inference.

## Availability and Requirements

**STARNET 2 **and the associated **HEATSEEKER **module are freely available on the Web, and do not require user registration: 

## List of Abbreviations

GO: Gene Ontology; GEO: Gene Expression Omnibus.

## Authors' contributions

DJ and VV designed the project, coded the Web interface, and wrote the manuscript. DJ collected and curated microarray data from GEO, analyzed the data, and coded the application logic. HC collected and curated microarray data from experiments conducted on human Affymetrix arrays. All authors read and approved the final manuscript.
